# Physiological and Biomechanical Evaluation of a Training Macrocycle in Children Swimmers

**DOI:** 10.3390/sports7030057

**Published:** 2019-03-04

**Authors:** Sara Ferreira, Diogo Carvalho, Ana Sofia Monteiro, José Arturo Abraldes, João Paulo Vilas-Boas, Argyris Toubekis, Ricardo Fernandes

**Affiliations:** 1 Centre of Research, Education, Innovation and Intervention in Sport, Faculty of Sport, University of Porto, 4200-450 Porto, Portugal; sara_ferreira_1120@hotmail.com (S.F.); diogoduarte_03@hotmail.com (D.C.); a.sofia.monteiro@gmail.com (A.S.M.); jpvb@fade.up.pt (J.P.V.-B.); ricfer@fade.up.pt (R.F.); 2 Porto Biomechanics Laboratory, University of Porto, 4200-450 Porto, Portugal; 3 Faculty of Sport Science, University of Murcia, 30720 San Javier, Spain; abraldes@um.es; 4 School of P.E. & Sport Science, National and Kapodistrian University of Athens, 17237 Athens, Greece

**Keywords:** performance, lactate, stroke rate, stroke length, stroke index, macrocycle

## Abstract

Physiological responses related to 400-m front crawl performance were examined in a 11-week training macrocycle in children 11.6 ± 1.2 years old. Fourteen girls and twenty-nine boys completed a maximum intensity 400-m test, at the beginning (Τ1) and at the end of four weeks of general preparation (Τ2), four weeks of specific preparation (Τ3), and three weeks of the competitive period (Τ4). Blood lactate (La), blood glucose (Glu) and heart rate were measured post effort. Stroke rate (SR), stroke length (SL) and stroke index (SI) were measured during the test. The 400-m time was decreased at T2, T3, and T4 compared to T1 by 4.2 ± 4.9, 7.5 ± 7.0, and 8.6 ± 7.3% (*p* < 0.05) and at T3 and T4 compared to T2 by 3.1 ± 4.3 and 4.2 ± 4.6%, respectively (*p* < 0.05). La was not different between tests (*p* > 0.05) and Glu was decreased at T3 compared to other testing moments (*p* < 0.05). SR, SL, and SI were higher at T3 and T4 compared to T1 (*p* < 0.05). SL and SI were also increased at T4 compared to T2 (*p* < 0.05). Performance changes from T1 to T2 were related to SL and SI changes (r = 0.45 and 0.83, *p* < 0.05), and subsequent changes between T2 to T3 were related to SR, SI, La, and Glu changes (r = 0.48, 0.68, 0.34, and 0.42, *p* < 0.05). Performance change from T3 to T4 was related to SL, SI, and La modifications (r = 0.34, 0.70, and 0.53, *p* < 0.05). Performance gains may be related to various biomechanical or physiological changes according to training macrocycle structure.

## 1. Introduction

Understanding the multiple factors affecting swimming performance in children may help in developing appropriate training plans that may facilitate long-term success at a later age. A well-designed year plan is a key factor for competitive performance, and this should be effectively applied in early age [1,2]. The year-plan is normally divided into shorter periods, the macrocycles. Performance changes occurring within a year-plan or a macrocycle in child swimmers may be attributed to a combination of factors such as anthropometry, biomechanics/technique, hydrodynamic, and physiological/energetic changes that may be critical in some training periods but not in others depending on the periodization applied [3,4,5,6].

Previous research in swimming training including children and young athletes focused mainly on short distance performance relative to changes in anthropometry, hydrodynamic, biomechanical/coordinative or energetic variables (25–100 m) [3,4,7,8,9]. The findings of these studies highlight the importance of the hydrodynamic factors changes (such as active and passive drag relative to training periodization [7]), despite a great intra- and inter-swimmer variation in technical variables [4]. However, no significant changes were observed in kinematic variables such as stroke rate (SR) and stroke length (SL) despite improvement of 100-m performance in female swimmers following 11 weeks of training [9]. Furthermore, improvement of physiological variables, such as maximum oxygen uptake and lactate concentration (La), was accompanied by improved performance in the 100-m test [8].

In addition to the previous research in short distance performance, the 400-m test has attracted attention in swimming research [10,11,12,13], probably because during a maximum 400-m effort, swimmers normally attain their maximum oxygen uptake with this distance being used for the aerobic power evaluation [10,11,12,13,14]. It is expected that both energetic and biomechanical factors contribute to 400-m performance depending on the training period content [10,11]. Moreover, cardiovascular adaptations are starting in early childhood [15], which may be related to fitness level and performance. Previous studies elaborate and quantify the importance of anthropometry, energetic, and biomechanical variables previously reported to be significantly related to 400-m performance in boy and girl swimmers [10,11,12,13]. However, the contribution of the biomechanical, anthropometric, and energetic variable was examined over a long duration period of two years [12,13]. Then, a significant maturation effect may have masked changes that occur within a short-term training period. It would be helpful for coaches and scientists to better understand metabolic and biomechanical changes occurring within a macrocycle preparation that may be connected to 400-m performance. Normally, the training content varies between training periods, enhancing technical ability in the preparatory period and metabolic stress in the competitive period [6,7,10,11].

Therefore, the purpose of the current study was to examine the physiological and biomechanical responses related to 400-m swimming performance in an 11-week training macrocycle in children aged 10–14 years old.

## 2. Materials and Methods

Forty-three swimmers (14 girls and 29 boys) participated in the current study. Their chronological age and main characteristics of the participants are presented in Table 1. Their level of maturation was also obtained [16]. All swimmer parents/guardians were informed about the purpose and design of the study and signed an informed consent prior to commencement of the study, which was conducted in accordance with the declaration of Helsinki for research with human subjects and approved by the local University ethical committee (CEFADE 04-2017).

Within an 11-week training macrocycle, swimmers were tested in four different moments: At the beginning of the general preparation period (Τ1) and at the end of the four weeks of general preparation period (Τ2), four weeks of specific preparation period (Τ3), and three weeks of the competitive period (Τ4). The training content of each period is indicated in Table 2. During the examination period, swimmers maintained their regular daily school activities and they did not participate or compete in other sporting activities. Each testing moment included a maximum effort 400-m front crawl timed by qualified timekeepers (Seiko, Tokyo, Japan). The final 400-m time and that of each 100-m split were recorded. A video recorder was placed in the middle of the pool, above the water and perpendicular to the swimmer’s direction at 10 m distance away from the swimming line, recording each 400-m test. SR was calculated for each 25-m section by the time taken to complete three upper limbs cycles (the same observer for all evaluations, intraclass correlation coefficient: 0.99; Kinovea software 8.15, Open source project, Bordeaux, France). SL was calculated by the quotient of mean speed with the mean SR and stroke index (SI) was determined as the product of mean speed with the mean SL.

Heart rate was measured using telemetry during the recovery period immediately after the 400-m test with the 10, 30, 60, and 120 s values recorded and used for data analysis (Polar Electro, Kempele, Finland). During the third minute of recovery after the completion of the 400-m test, a capillary blood sample was collected from a finger to measure La (Lactate Pro, Arkay Inc., Kyoto, Japan). One more capillary blood sample was collected from a different finger to measure glucose (Glu) levels (Glucocard™ Mx, A. Menarini, Paço de Arcos, Portugal). At the end of the 400-m test, swimmers indicated the rating of perceived exertion (RPE) in a 20-point scale [17].

### Statistical Analysis

Normal distribution was tested using the Kolmogorov–Smirnov test, and sphericity was verified using Mauchly’s test. When the assumption of sphericity was not met, the significance of F-ratios was adjusted according to the Greenhouse–Geisser procedures. One-way analysis of variance (ANOVA) on repeated measures was used to compare SR, SL, SI, La, and Glu (four testing periods). Two-way ANOVA was used to compare heart rate recovery (four testing periods x four points of recovery) and changes on swimming time, SR, SL, and SI in each 100-m lap in the 400-m test. A Tukey honest significance difference post-hoc test was used to compare means when significant F-rations were found. Pearson’s correlation coefficient was used to examine relations between variables. Data are presented as mean ± standard deviation (SD). Statistical significance was set at *p* < 0.05.

## 3. Results

### 3.1. Changes in 400-m Time, Stroke Rate, Stroke Length, Stroke Index, and Anthropometry

**Figure 1:**
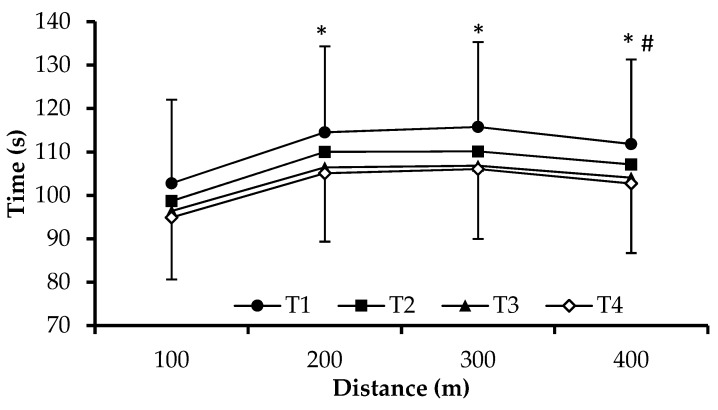
Time for each 100-m split in the 400-m front crawl tests at testing moments T1 (beginning of the general preparation period), T2, T3, and T4 (end of four weeks of general preparation period, four weeks of specific preparation period, and three weeks of the competitive period). *: *p* < 0.05 compared to 100 m, #: *p* < 0.05 compared to 200 and 300 m. (Mean ± SD, n = 43).

**Figure 2:**
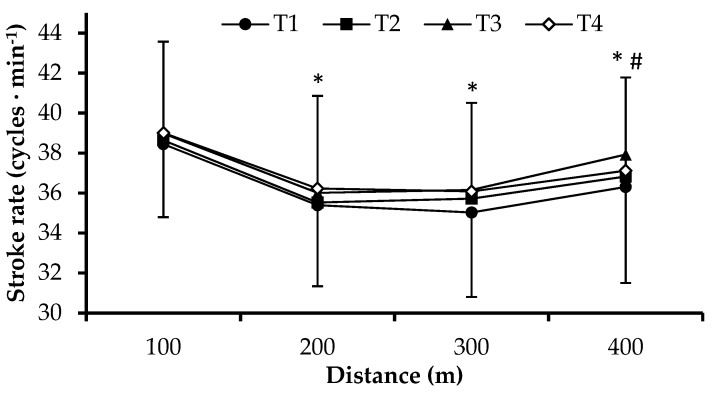
Stroke rate in each 100-m split of the 400-m front crawl tests completed at testing moments T1 (beginning of the general preparation period), T2, T3, and T4 (end of four weeks of general preparation period, four weeks of specific preparation period, and three weeks of the competitive period). *: *p* < 0.05 compared to 100 m, #: *p* < 0.05 compared to 200 and 300 m. (Mean ± SD, n = 43).

Swimmers were progressively increasing their performance across testing moments (F_3,126_ = 36.0, *p* = 0.00, Table 3). Time of the 400-m maximum front crawl effort was decreased after T2, T3, and T4 compared to T1 by 4.2 ± 4.9, 7.5 ± 7.0 and 8.6 ± 7.3%, respectively (*p* < 0.05, Table 3). Similarly, 400-m time was decreased in T3 and T4 compared to T2 by 3.1 ± 4.3 and 4.2 ± 4.6%, respectively. However, no difference was observed between T3 compared to T4 performance (Table 3). Time of each 100-m split in the 400-m test showed a similar trend across testing moments (Figure 1). SR was increased at T3 and T4 compared to T1 (F_3,126_ = 3.9, *p* = 0.01, Table 3) and presented the same trend in each 100-m split of the 400-m test (Figure 2). SL was increased after T2, T3, and T4 compared to T1 and after T4 compared to T2 (F_3,126_ = 14.16, *p* < 0.05, Table 3). Similarly, SI was increased after T2, T3, and T4 compared to T1 and after T4 and T3 compared to T2 (F_3,126_ = 42.53, *p* < 0.05, Table 3).

### 3.2. Physiological Responses and Rating of Perceived Exertion Following 400-m

**Figure 3:**
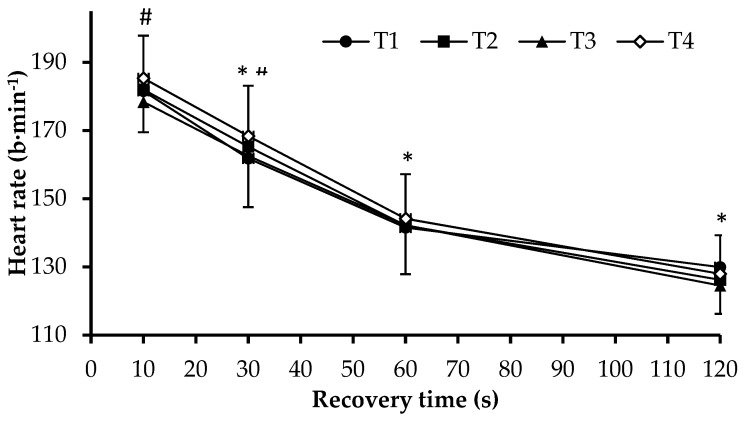
Heart rate changes during recovery after 400-m front crawl in testing moments T1 (beginning of the general preparation period), T2, T3, and T4 (end of four weeks of general preparation period, four weeks of specific preparation period, and three weeks of the competitive period). *: *p* < 0.05 compared to previous and next time point. #: *p* < 0.05 between T3 and T4. (Mean ± SD, n = 43).

Heart rate was no different between testing moments (T1, T2, T3, and T4, F_3,126_ = 1.49, *p* = 0.22), but it was decreasing after 10 to 20 s, from 20 to 30 s and 30 to 60 s of recovery in all periods (T1, T2, T3, and T4). An interaction between testing moments and recovery time was observed, showing a higher heart rate at 10 and 30 s recovery in T4 compared to T3 and T1 periods, respectively (F_9,378_ = 2.6, *p* = 0.01, Figure 3). La was no different between T1, T2, T3, and T4 testing moments (F_3,126_ = 1.27, *p* = 0.28, Table 3). Glu was decreased after T3 and T4 compared to both T1 and T2. Moreover, Glu was increased after T4 compared to T3 (F_3,126_ = 15.5, *p* = 0.00, Table 3). La was related to blood Glu in all testing moments (T1, r = 0.68, *p* < 0.01; T2, r = 0.49, *p* = 0.01; T3, r = 0.31, *p* = 0.05; T4, r = 0.49, *p* = 0.01). RPE was no different between testing moments (F_3,126_ = 0.14, *p* = 0.94, Table 3).

### 3.3. Correlations between Variables

In each testing moment, the mean 400-m speed was correlated with anthropometric, physiological, and biomechanical variables (Table 4). Correlations between mean 400-m speed change and respective changes in La, Glu, SR, SL, and SI at testing moments T2 vs. T1, T3 vs. T2, T4 vs. T3, and T4 vs. T1 are shown in Table 5.

## 4. Discussion

The purpose of the current study was to examine changes in physiological and biomechanical characteristics relative to 400-m front crawl performance during an 11-week macrocycle in child swimmers. A 9% performance improvement was related more with technical/biomechanical characteristics in the general and specific preparation and to metabolic changes in the competitive period. In previous studies with children, boy and girl swimmers reported 400-m performance improvement of 2–3% within a year of training [12,13], which is lower compared to the value of 9% observed in the current study. However, a similar improvement of 8% has been reported in 100-m performance after a year of training in high level 11- to 12-year-old child swimmers [4]. Other studies have reported a 2% improvement in the 100-m test within a short 5-week period of training in 11-year-old boys and girls [8], and 3% improvement in the same distance following eleven weeks of training period in 13-year-old female swimmers [9].

Current study swimmers started training at a lower performance level compared to swimmers participated in previous studies (best 400-m time 7:24 and 6:13 min:s, respectively) [12,13] and possibly may had higher likelihood for improvement. Whatever the case, performance improvement was progressive from T1 to T2 and from T2 to T3, but no improvement was observed between T3 and T4. The short duration of the competitive period and lack of a taper may have reduced the likelihood of further improvement in the last training stage of the study. In fact, in the competitive period, swimmers completed three weeks with increased distance of anaerobic training compared to previous periods, aiming inducing adaptations related to competitive distance that were in most of the cases shorter than 400 m (i.e., mostly 50 and 100 m). Then, it is likely that less attention was given to aerobic training, which is the major component of a 400-m swimming distance [18]. Unchanged performance in a 400-m front crawl test and maintenance of aerobic contribution at the end of competitive period compared to specific training period has been observed in young swimmers [10]. With this scenario, the aerobic adaptations of the swimmers were maintained from T3 to T4, and the 400-m performance was not improved significantly.

All tested variables were correlated with the 400-m front crawl performance in each testing moment, confirming the importance of anthropometric, biomechanical, and physiological variables for this event in child swimmers [11,12,13]. It is interesting that SI, a recognized practical and valid index of technical efficiency that improves with swimming training progression [5,7], showed the best correlation with the 400-m speed. It should also be considered that SI was a significant determinant of performance change in all testing moments, which is in agreement with previous findings indicating that the periodization structure affects the importance of various variables on competitive performance [4,6]. It is interesting to note that improvement of 400-m performance in the current study was achieved in a very short period compared to the literature, while comparable improvements were observed following long duration training cycles (i.e., 1–2 years) [11,12,13]. It seems that appropriate planning of training to improve technique, as was the case in the general preparation training cycle in the current study, facilitated technical improvement enhancing SI and SL. The relative significance of technique on a 400-m test performance following the general preparation period has confirmed previous data [10].

Whatever the case, improvement of performance from T1 to T2, T2 to T3, and T3 to T4 was achieved with the support of different variables. It was the contribution of SL in the general preparation (T1 to T2) and the contribution of SR in the specific preparation (T2 to T3) that were related to performance changes. In the competitive period (T3 to T4), it was the metabolic component that contributed more, with La increments relating to performance changes. This is in agreement to previous studies showing increased support from the glycolytic energy system following the competitive period in a 400-m test [10]. Increased training intensity may facilitate anaerobic adaptations in children, and this is reflected with a greater rate of lactate accumulation [8]. La was not increased from T1 to T4, but there was a strong correlation between La changes and performance changes from T3 to T4 at the end of the competitive period. The observed correlation of La changes with performance modifications for T3 to T4 despite performance not being significantly improved may indicate that anaerobic contribution has no critical impact in efforts of 6–7 min duration in children as it has been observed in young swimmers (i.e., anaerobic contribution 12%) [11].

Decreased levels of Glu following the 400-m test were observed after the specific preparation period (T3) compared to all other testing moments and after T4 compared to T2. A previous study reported unchanged Glu with increasing intensity swimming before and after a period of 10-week training, despite swimmers improving their endurance within this period [19]. Others reported increased Glu after a maximum 200-m test compared to submaximal intensity swimming [20]. Glu levels are the product of glucose release and glucose uptake and may vary with training intensity and carbohydrate diet content [21]. During the specific preparation period, training intensity may have increased the energy demands and glucose uptake from the circulation may have been facilitated, showing decreased levels at the end of T3. Whatever the case, it seems that Glu levels do not reflect the exercise intensity per se, but increased Glu from T2 to T3 was related to performance enhancement, indicating that a supporting blood substrate status could be beneficial for performance improvement in a 400-m test. The effect of Glu on performance needs more attention in future studies, particularly through including well controlled diet content.

A fast heart rate recovery 1 min following an exercise test is connected with improved endurance [22], but no changes in this variable were observed between the four testing moments. It was only the maximum heart rate that increased after the 400-m test in the last testing moment (after the competitive training period, T4). It has been found that children’s heart responds to training-induced adaptations decreasing the resting heart rate, but there is no evidence concerning maximum heart rate modifications [14]. The increased maximum heart rate observed in the last testing moment (T4) may be attributed to the increased absolute effort exerted by the swimmers, facilitated by the better readiness to compete at the end of the competitive period compared to previous testing moments.

## 5. Conclusions

The current data confirm the importance of physiological and technical components development in child swimmers. A well-designed 11-week training macrocycle may lead to substantial performance improvement, attributed to technical–biomechanical and physiological factors. During the general preparation period, SL changes may be more important, while metabolic changes may facilitate performance improvement during specific preparation and competitive periods. SI improvement is important all over the training macrocycle. It is suggested that emphasis on technique development should be the core of competitive preparation in this age-group of swimmers. Moreover, short periods of training may be used to stress energy systems and enhance performance. The current finding highlights the importance of combined physiological and biomechanical data gain for child swimmer evaluation.

## Figures and Tables

**Table 1:** Anthropometric characteristics of young swimmers that participated in the current study.

Measured Variables	Boys (n = 14)	Girls (n = 29)	Total Sample (n = 43)
Chronological age (years)	11.90 ± 1.08	10.74 ± 0.91	11.62 ± 1.19
Body mass (kg)	45.02 ± 9.27	40.36 ± 8.43	43.50 ± 9,17
Stature (m)	1.53 ± 0.10	1.46 ± 0.09	1.51 ± 0.10
Tanner stage	2.93 ± 0.95	2.71 ± 1.15	2.86 ± 1.02
Training sessions per week	5.79 ± 0.62	5.43 ± 1.16	5.67 ± 0.84

**Table 2:** Swimmers training content expressed as the percentage of distance covered within each period of the macrocycle.

Training Type	General Preparation Period	Specific Preparation Period	Competitive Period
Aerobic training	96%	93%	84%
Anaerobic training	4%	7%	16%
Technical training	32%	31%	21%

**Table 3:** Changes in body mass, stature, 400-m time front crawl, blood lactate, blood glucose, rating of perceived exertion, mean stroke rate, mean stroke length, and mean stroke index during the four testing moments. (Mean ± SD, n = 43).

Measured Variables	T1	T2	T3	T4
Body mass (kg)	43.50 ± 9.17	44.11 ± 9.20	43.90 ± 8.92	44.67 ± 9.35 *
Stature (m)	1.51 ± 0.10	1.52 ± 0.11 *	1.53 ± 0.11 *	1.54 ± 0.10 *^+^
Time 400-m (s)	444.40 ± 76.95	426.00 ± 67.61 *	412.83 ± 61.11 *^+^	408.95 ± 61.40 *^+^
Blood lactate (mmol∙L^−1^)	5.97 ± 2.37	6.47 ± 3.06	5.72 ± 2.16	6.24 ± 2.56
Blood glucose (mmol∙L^−1^)	109.05 ± 16.02	111.02 ± 16.42	93.33 ± 16.99 *^+^	101.30 ± 19.61 *^+×^
Rate of perceived exertion	14.88 ± 1.95	14.88 ± 2.04	14.74 ± 2.22	14.95 ± 2.32
Stroke rate (cycles∙min^−1^)	36.3 ± 3.9	36.7 ± 4.3	37.3 ± 4.4 *	37.1 ± 4.4 *
Stroke length (m∙cycle^−1^)	1.541 ± 0.242	1.587 ± 0.237 *	1.607±0.241 *	1.632 ± 0.226 *^+^
Stroke index (m^2^∙s^−1^∙cycle^−1^)	1.455 ± 0.451	1.553 ± 0.448 *	1.615 ± 0.451 *^+^	1.656 ± 0.442 *^+^

T1: Beginning of the general preparation period. T2: End of four weeks of general preparation period. T3: End of four weeks of specific preparation period. T4: End of three weeks of the competitive period. *: *p* < 0.05 compared to T1, +: *p* < 0.05 compared to T2, ×: Compared to T3.

**Table 4:** Correlation coefficient (and respective *p*-value) for the relationships between mean 400-m swimming speed in each testing moment and respective body mass, stature, mean stroke rate, mean stroke length, mean stroke index, blood lactate, and blood glucose. All correlations are statistically significant (*p* < 0.05).

400-m Speed	Body Mass	Stature	Stroke Rate	Stroke Length	Stroke Index	Blood Lactate	Blood Glucose
T1	0.36 (0.02)	0.46 (0.01)	0.48 (0.01)	0.77 (0.00)	0.93 (0.00)	0.46 (0.01)	0.45 (0.01)
T2	0.36 (0.02)	0.48 (0.01)	0.54 (0.01)	0.70 (0.00)	0.92 (0.00)	0.40 (0.01)	0.51 (0.01)
T3	0.38 (0.02)	0.48 (0.01)	0.46 (0.01)	0.67 (0.00)	0.91 (0.00	0.38 (0.02)	0.58 (0.01)
T4	0.34 (0.04)	0.43 (0.01)	0.61 (0.01)	0.65 (0.00)	0.92 (0.00)	0.64 (0.01)	0.60 (0.01)

T1: Beginning of the general preparation period. T2: End of four weeks of general preparation period. T3: End of four weeks of specific preparation period. T4: End of three weeks of the competitive period.

**Table 5:** Correlation coefficient (and respective *p*-value) of speed changes in testing moments T1–T2, T3–T2, T4–T3, and T4–T1 with the corresponding changes in mean stroke rate, mean stroke length, mean stroke index, blood lactate, and blood glucose.

400-m Speed	Stroke Rate	Stroke Length	Stroke Index	Blood Lactate	Blood Glucose
T2–T1	0.25 (0.11)	0.45 (0.01) *	0.83 (0.00) *	0.18 (0.24)	0.03 (0.82)
T3–T2	0.48 (0.01) *	0.20 (0.19)	0.68 (0.00) *	0.34 (0.03) *	0.42 (0.01) *
T4–T3	0.19 (0.21)	0.34 (0.02) *	0.70 (0.00) *	0.53 (0.01) *	0.15 (0.33)
T4–T1	0.17 (0.29)	0.56 (0.00) *	0.87 (0.00) *	0.36 (0.02) *	0.34 (0.03) *

T1: Beginning of the general preparation period. T2: End of four weeks of general preparation period. T3: End of four weeks of specific preparation period. T4: End of three weeks of the competitive period. *: Indicates significant correlation.
